# Swine Colibacillosis: Analysis of the Gut Bacterial Microbiome

**DOI:** 10.3390/microorganisms12061233

**Published:** 2024-06-19

**Authors:** Wanli Sha, Emad Beshir Ata, Man Yan, Zhijie Zhang, Honggang Fan

**Affiliations:** 1 Heilongjiang Key Laboratory for Laboratory Animals and Comparative Medicine, College of Veterinary Medicine, Northeast Agricultural University, Harbin 150030, China; jlshawanli@163.com; 2 Technology Innovation Center of Pig Ecological Breeding and Disease Prevention and Control, College of Animal Science and Technology, Jilin Agricultural Science and Technology University, Jilin 132109, China; jlyanjingyu@163.com; 3 Parasitology and Animal Diseases, Veterinary Research Institute, National Research Centre, Dokki, Cairo 12622, Egypt; emadvet2003@yahoo.com; 4 Heilongjiang Provincial Center for Disease Control and Prevention, Harbin 150030, China; lxz0451@126.com

**Keywords:** colibacillosis, enterotoxigenic, microbiome, histopathology, *Bacteroidetes*, *Firmicutes*

## Abstract

This study aimed to evaluate the disruption of the swine gut microbiota and histopathological changes caused by infection with enterotoxigenic *E. coli*. Fecal samples were collected from piglets suffering from diarrhea post-recovery and healthy animals. Intestinal tissues were collected for histopathological changes. The results revealed histopathological changes mainly in the ileum of the infected animals compared to those in the ileum of the control and recovered animals. The operational taxonomic units (OTUs) revealed that the *E. coli* diarrheal group exhibited the highest bacterial richness. Principal coordinate analysis (PCoA) corroborated the presence of dysbiosis in the gut microbiota following *E. coli*-induced diarrhea. While the normal control and infected groups displayed slight clustering, the recovery group formed a distinct cluster with a distinct flora. *Bacteroidetes*, *Firmicutes*, and *Fusobacteria* were the dominant phyla in both the healthy and recovered piglets and in the diarrheal group. LEfSe and the associated LDA score analysis revealed that the recovered group exhibited dominance of the phyla *Euryarchaeota* and *Bacteroidota*, while groups N and I showed dominance of the phyla *Firmicutes* and *Fusobacteriota*, respectively. The LDA scores highlighted a significant expression of the *Muribaculacea* family in group R. The obtained findings will help in understanding the microbiome during swine colibacillosis, which will support control of the outbreaks.

## 1. Introduction

The importance of swine has been clarified not only because they are a main protein source but also because they can be used as models for studying many human diseases. Furthermore, they are vessels for many zoonotic pathogens [1]. The swine industry suffers from severe financial losses due to infection with many viral or bacterial pathogens [2,3,4,5].

Swine colibacillosis caused by Gram-negative *Escherichia coli* (*E. coli*) is an epidemiological threat not only to the pork industry but also to human health due to the zoonotic importance of some virulent strains [6]. It is the cause of multiple symptoms, including polyserositis, septicemia, edema disease (ED), postweaning diarrhea (PWD), neonatal diarrhea, and urinary tract infection [7]. It has a significant impact because of severe financial losses in the form of decreased body weight, reduced weight gain, and increased treatment and vaccination costs [6]. The average mortality rates due to PWD were 9.4 to 12.6%, while in acute outbreaks, a percentage of up to 20 to 30% over a 1- to 2-month time span was recorded [8].

Although *E. coli* is a normal commensal gastrointestinal inhabitant of many animals, including pigs, it might cause local or systemic disease in different animal species with zoonotic importance to humans. Pathotypes of *E. coli* strains that express specific virulence traits tend to cause diarrhea [9]. The main pathotypes of *Escherichia coli* include enteropathogenic (EPEC), enterohaemorrhagic (EHEC), enteroaggregative (EAEC), enterotoxigenic (ETEC), enteroinvasive (EIEC), vero- or Shiga-like toxin-producing (VTEC or STEC), and diffusely adherent (DAEC) pathogens. Among these, the fimbriae F4 (K88) and F18 of ETEC are the most pervasive etiological agents that cause postweaning diarrhea (PWD) in pigs [10,11].

Symptoms of colibacillosis might appear in the same outbreak or later, either concurrently or separately [11]. Shortly after weaning, piglets become more vulnerable to microbial infections because they are deprived of immunoglobulin-rich sow milk after weaning. This critical period is typically related to the drastic increase in intestinal *E. coli* infection, which is characterized by abrupt mortality or extreme diarrhea [7].

The gastrointestinal tract is a complex ecosystem that has a large number of microorganisms with multiple metabolic activities together with their genes microbiome [12]. It varies with age, diet, and other numerous factors present in the intestines of pigs. This community shifts along the intestinal tract segments. The intestinal microbiota of pigs begins to colonize from birth to weaning [13]. Therefore, weaning stress and dietary modifications may easily disturb the gut microflora of young pigs, increasing their susceptibility to pathogenic bacteria [14].

Despite being among the initial bacteria that settle in young pigs’ intestines at birth, *E. coli* gradually disappears after weaning. The intestinal microbiota may be impacted by the presence of ETEC infection or an increase in *E. coli* abundance during the postweaning period [15]. The jejunum and feces of weaned pigs had lower *Bacteroidetes*:*Firmicutes* ratios and microbial diversity due to ETEC K88 infection [16]. The two most common intestinal microbial phyla in the guts of young pigs are *Firmicutes* and *Bacteroidetes*, which work together to utilize carbohydrates [16]. As a biomarker for intestinal dysbiosis, a decreased fecal *Bacteroidetes*:*Firmicutes* ratio was reported in pigs suffering from other diarrheal illnesses [17]. Significant alterations in the community structure have been reported in association with numerous cases of enterotoxigenic K88 or F18 infections. An increase in the relative abundance of the *Proteobacteria* family in the colon or ileum due to the increase in the abundance of *Escherichia coli* or *Helicobacteraceae* has been observed in pigs exposed to enterotoxigenic K88 or F18 [18,19]. When post-suckling pigs were infected with enterotoxigenic F18, the proportion of *Lactobacillus* in the ileum decreased. The disruption of the intestinal microbiota caused by ETEC infection decreases the generation of volatile fatty acids in the colon and further modifies the intestinal ecology to favor pathogen growth [20,21]. Numerous microbiota alterations have been found to be inversely related to the general gut health and growth performance of weaned pigs. Accordingly, the present study aimed to evaluate the disruption of the swine gut microbiota due to infection with enterotoxigenic *E. coli* (ETEC) and to determine histopathological changes.

## 2. Materials and Methods

### 2.1. Ethics Statement

All animal experiments and test operations involved in the current study were approved and followed up by the Laboratory Animal Research Ethics Committee (SRM-11) of Northeast Agricultural University (protocol number NEAUEC2023 04 78).

### 2.2. Animals and Fecal Sample Collection

Changbai piglets from a farm located in Jilin Province, China (125°40′ N, 42°31′ E), were used for sample collection in this study. Thirty fresh fecal samples were collected from 30 piglets suffering from colibacillosis-induced diarrhea (I) one month after recovery (R) to track changes in the gut microbiota of the piglets. Ten fresh fecal samples were collected from healthy animals for use as controls (N).

All samples were collected, transported to the laboratory on dry ice, and stored at −80 °C until processing.

### 2.3. Histopathology

Small intestinal tissue samples were processed as previously described [22]. Briefly, the collected small intestine samples were fixed in 4% paraformaldehyde, trimmed, and washed under running water to remove formaldehyde. The tissues were dehydrated in alcohol with a gradient concentration from low to high, followed by soaking in xylene to make them transparent. Then, the tissues were embedded in wax blocks in liquid paraffin and cut into 3 μm slices. The tissue sections were soaked in xylene, treated with high- to low-concentration gradient alcohol, washed with water, soaked with hematoxylin–eosin stain, and then washed with water, alcohol, and light ammonia. The stained tissue sections were dried and sealed with neutral resin, followed by microscopic examination (Leica, Wetzlar, Germany). The bright-field images were obtained by three experienced examiners independently. The three observers were blinded to the tissue sources and processing information [23].

### 2.4. Molecular Identification

#### 2.4.1. DNA Extraction

Fecal DNA extraction was carried out according to the instruction manual (EasyPure^®^ Stool Genomic DNA Kit, TransGen Biotech, Beijing, China). The purity and quality of the eluted fecal DNA samples were measured using an ultramicro-UV–vis spectrophotometer (Pono-550, Porabio, Zhejiang, Hangzhou, China). All the eluted DNA samples were preserved at −20 °C until further processing.

#### 2.4.2. Polymerase Chain Reaction Amplification and Sequencing

According to the concentration of the eluted DNA, a total of 20 ng of DNA was used for polymerase chain reaction. Based on the variable region of 16S rDNA V3-V4, the primers in Table 1 were used for PCR.

The PCR mixture was performed in a total volume of 50 μL consisting of 25 μL of PrimeSTAR Max Premix (2×) (TaKaRa, Dalian, China), 20 ng of template DNA, 0.75 μL of each primer (10 μM), and sterile water to adjust the volume of the mixture. The PCR thermal conditions were as follows: initial denaturation at 98 °C for 2 min; 25 cycles of denaturation at 98 °C for 30 s; annealing at 50 °C for 30 s; extension at 72 °C for 1 min; and a final extension at 72 °C for 5 min and holding at 4 °C. The obtained amplicons were visualized by electrophoresis using a stained 1% agarose gel [24]. All of the obtained amplicons were subjected to sequencing at Allwegene Company (Beijing, China) using the Illumina MiSeq PE300 platform (Illumina, Inc., San Diego, CA, USA).

### 2.5. Analysis of the Gut Microbial Composition

The raw data were spliced and quality filtered, and then, the sequences were clustered at the 97% similarity level. Species annotation, phylogenetic, and taxonomic analyses of the bacterial 16S rRNA gene were performed using RDP Classifier (version 2.2), which is available at https://bioweb.Pasteur.fr/packages/pack@rdp_classifier@2.2 (accessed on 5 February 2024). Mothur (V 1.3) software https://mothur.org/wiki/mothur_v.1.3.0/ (accessed on 10 February 2024) was used for alpha diversity analysis (mainly Chao1, Ace, Shannon, and Simpson indices, species accumulation curves, and Shannon curves).

Beta diversity analysis was performed based on binary Jaccard, Bray–Curtis, and unweighted UniFrac algorithms, and R (version 4.2.6) was used for visualization. Finally, linear discriminant analysis (LDA) was used to estimate the abundance of each component (species). Species with differences in sample composition between the two groups were identified using meta-statistical analysis.

### 2.6. Statistical Analysis

GraphPad Prism (version 9) was used for the statistical tests. The statistical significance of differences was calculated with one-way ANOVA. *p* < 0.05 was considered significant, and ns was considered nonsignificant. * *p* < 0.05; ** *p* < 0.01;*** *p* < 0.001, and ns *p* > 0.5.

## 3. Results

### 3.1. Histopathological Changes in the Small Intestine

**Figure 1:**
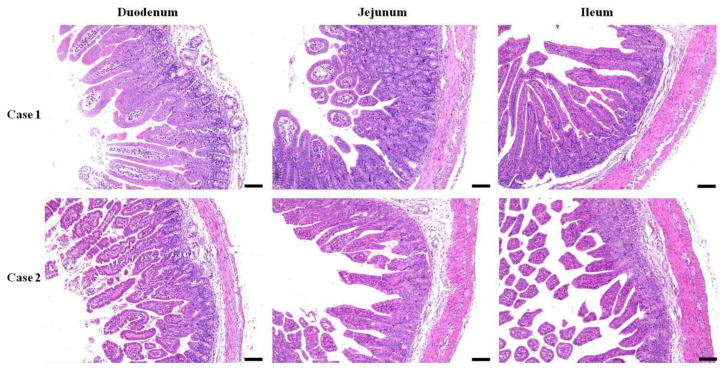
Histopathological changes in the small intestine segments. Duodenal, jejunal, and ileal samples of piglets infected with *E. coli* from pig farm were examined in pathological tissue sections and with HE staining (Bar = 100 µm).

The morphology of the three segments of the small intestine from both the *E. coli*-infected and control recovery groups was assessed. Generally, the tissues obtained from infected piglets displayed anomalies such as swelling of the lamina propria and submucosa in both duodenal and jejunal tissues, in contrast to those from the control group, along with edema of the lamina propria in the ileal tissue and neutrophil infiltration (Figure 1). Villous atrophy accompanied by crypt hyperplasia was the predominant lesion observed. An atrophic pattern was cleared in the mucosa of the duodenum, jejunum, and ileum in diarrheal piglets infected with *E. coli*. The severity of atrophy varied among different segments of the small intestine, mainly in the ileum and villi, leading to severe disruption of the villous structure. Additionally, both the villus length and width were greater in the infected group than in the control group. Mild epithelial lesions were noted at the villi tips and were often associated with villous atrophy. Damage to the crypts of the Lieberkühn epithelium was also observed in the *E. coli*-infected group, with elongated and irregular crypts noted (Figure 1).

### 3.2. Alpha Diversity of Gut Microbes after E. coli Infection

**Figure 2:**
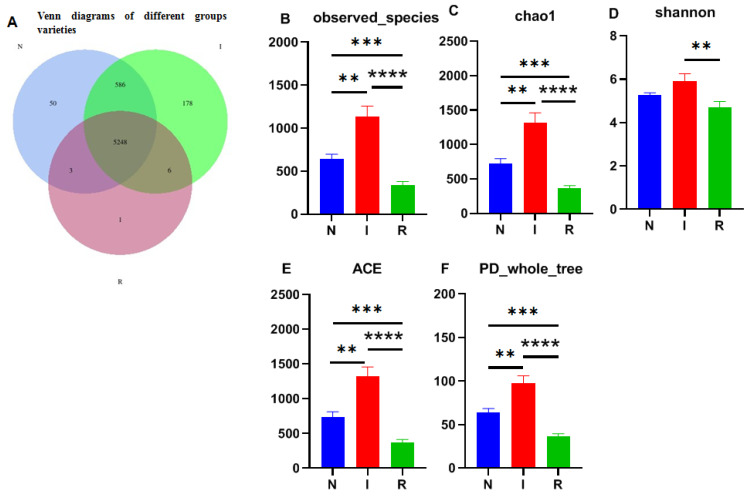
Venn map and alpha-diversity analysis. (**A**) Venn diagrams of different groups varieties. (**B**) Observed_species. (**C**) Chao1 observed number of species. (**D**) Shannon–Wiener index. (**E**) ACE. (**F**) PD_whole_tree. One-way ANOVA was employed for the statistical analysis (** *p* < 0.01; *** *p* < 0.001; **** *p* < 0.0001). Abbreviations: N, samples from normal control group; I, samples from the infected group; R, samples from recovery group.

The 16S rRNA sequencing resulted in millions of raw readings, yielding a total of 6072 operational taxonomic units (OTUs). Among these, group I exhibited the highest microflora composition with 6018 OTUs, while groups N and R had 5887 and 5258 OTUs, respectively. A significant difference in microflora composition between group I and the other two groups was recorded. Notably, 5248 OTUs were shared among all three groups (Figure 2A).

The observed species index (Figure 2B) and Chao1 index (Figure 2C) were utilized to assess microbial richness, while the Shannon–Wiener index (Figure 2D) was used to evaluate species diversity, collectively contributing to an α diversity evaluation. The Chao1 index revealed significant differences among all groups, with group I displaying notably greater strain richness than the other groups. Additionally, the observed species results indicated a substantial increase in the number of observed OTUs with deeper sequencing, which was particularly evident in groups I and N compared to group R, where group I exhibited the highest count. Moreover, the Shannon index highlighted greater microbial diversity in groups N and I than in group R, with greater diversity in group I. Overall, *E. coli* infection resulted in microbial translocation in the gut, impacting the microflora composition as invasion progressed. The ACE and PD whole-tree analyses further supported these findings, indicating significantly greater alpha diversity in group I than in groups N and R (Figure 2E,F).

### 3.3. Beta Diversity and Taxa of Bacteria in the Gut

**Figure 3:**
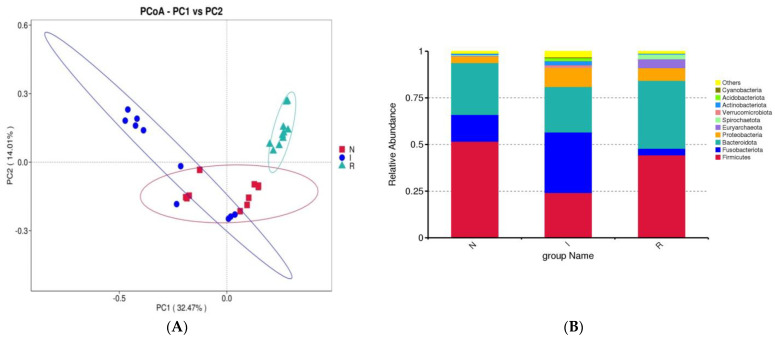

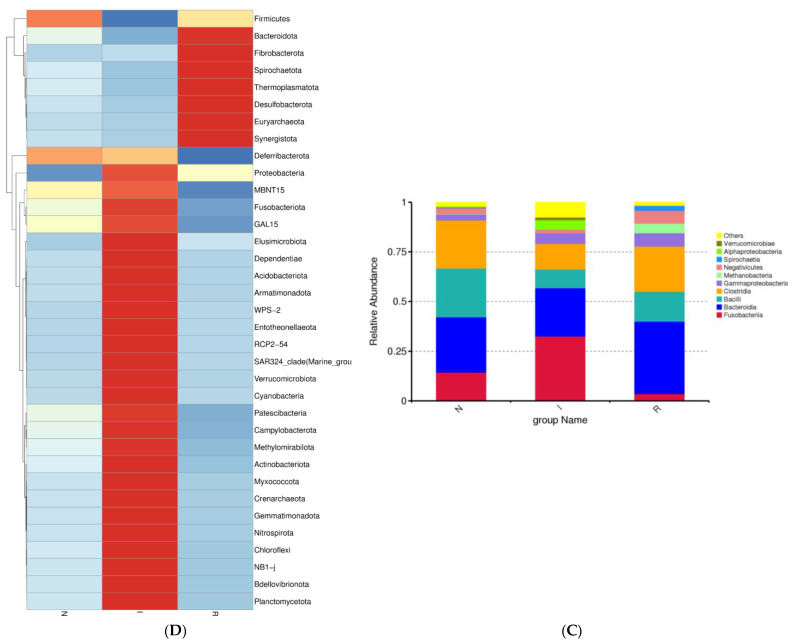
β-diversity and analysis of flora composition. (**A**) Beta diversity. (**B**) Analysis of flora composition at phylum level. (**C**) Analysis of flora composition at class level. (**D**) Heatmaps. Abbreviations: N, samples from normal control group; I, samples from the infected group; R, samples from recovery group.

Beta diversity was evaluated using principal coordinate analysis (PCoA) with weighted UniFrac distance matrices to demonstrate the resemblance among microbial communities. The PCoAs showed a slight similarity between groups N and I, while group R exhibited a clear differentiation from both (Figure 3A). To elucidate the impact of *E. coli* infection on the gut microflora composition, a detailed analysis at the phylum and class levels was carried out to delineate the taxonomic classification dynamics of microbes. At the phylum level, the dominant taxa in the gut microbial communities across all three groups were *Firmicutes*, *Fusobacteriota*, and *Bacteroidota*. In group N, *Firmicutes* accounted for 51.64% of the total bacteria, while *Fusobacteriota* constituted 14.37%. In group I, these percentages were 24.15% and 32.47%, respectively.

Conversely, in group R, the percentage of *Firmicutes* was 44.34%, and that of *Fusobacteria* was 3.55%. In comparison to group N, group I exhibited enrichments in *Fusobacteriota* and *Proteobacteria*, accompanied by a slight depletion of *Firmicutes*. Conversely, compared to group I, group R exhibited enrichment of *Firmicutes* and *Bacteroidota* but depletion of *Fusobacteriota* and *Proteobacteria* (Figure 3B).

A further analysis of the bacterial composition at the class level within the gut microbes was performed. In groups N, I, and R, the relative abundance of *Bacteroidia* represented 27.77%, 24.34%, and 36.40% of the population, respectively, indicating a noticeable increase during the recovery phase. In contrast, *Bacilli* constituted 24.58%, 9.42%, and 15.06% of the population in groups N, I, and R, respectively, indicating a notable decrease during the period of *E. coli* infection (Figure 3C). The heatmap depicted the expression levels of different bacterial compositions at the phylum level, with red indicating upregulated expression and blue indicating decreased expression. Notably, recovery group R exhibited significantly greater abundances of *Bacteroidota* and *Fibrobacterota*, in contrast to the distinct bacterial composition observed in the infected group (Figure 3D).

### 3.4. Taxonomic Cladogram and LDA Scores

**Figure 4:**
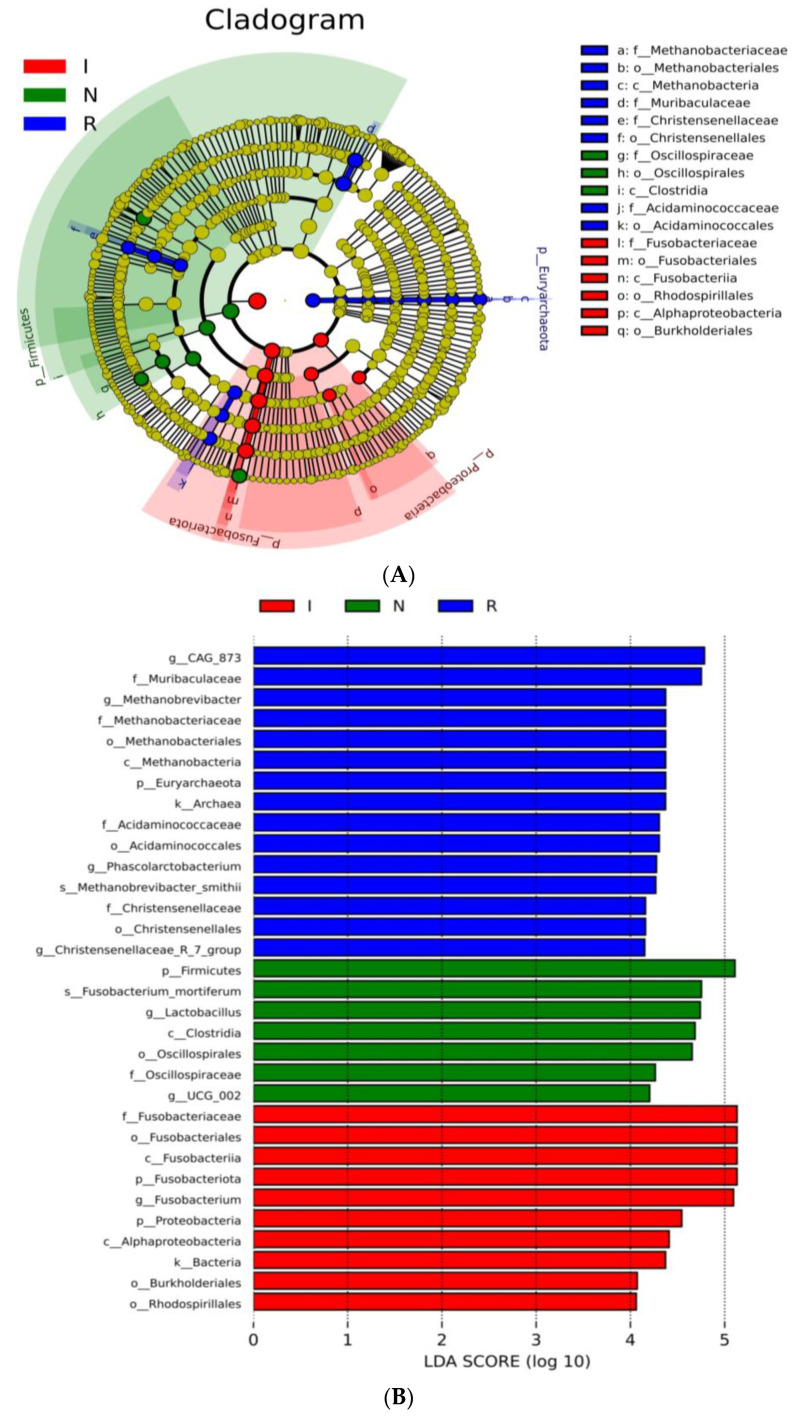
LEfSe analysis. (**A**) Cladogram of the LEfSe analysis of the gut microbiota in different groups. (**B**) Histogram of the LDA scores computed for features differentially abundant among N, I, and R piglets. LDA scores obtained from the LEfSe analysis of the gut microbiota in different groups. An LDA effect size of greater than 3 was used as a threshold for the LEfSe analysis. Abbreviations: N, samples from normal control group; I, samples from the infected group; R, samples from recovery group.

To comprehensively discern the disparities between the *E.* coli-infected group and the recovery group, LEfSe analysis of the gut microbial composition of the pigs was conducted. The taxonomic cladogram (Figure 4A) and the accompanying LDA scores facilitated the confirmation and visualization of the effects. The taxonomic cladogram derived from LEfSe analysis of sequences with a relative abundance ≥ 0.5% highlighted biomarker taxa through colored circles and shaded areas. The infected samples are shown in red; the normal samples are shown in green, and the recovery samples are shown in blue. The diameter of each circle reflects the abundance of that taxon within the community. Notably, the recovery group (R) exhibited dominance of the phyla *Euryarchaeota* and *Bacteroidota*, while groups N and I displayed dominance of the phyla *Firmicutes* and *Fusobacteriota*, respectively. LDA scoring was conducted with a threshold of log10 > 4 (Figure 4B). Interestingly, the LDA scores revealed pronounced expression of the *Lactobacillus*genusin group N; however, there were significantly greater LDA scores for the *Fusobacterium*genusin group I (Figure 4B). Furthermore, the LDA scores revealed pronounced expression of the *Muribaculacea*family, *Methanobrevibacter*, *Phascolarctobacterium,* and *Christensenellaceae* genera in group R, which are known for their anti-inflammatory functions (Figure 4B). LefSe revealed that the predominant residents in the gut bacterial communities varied distinctly among the three groups of pigs, consistent with the findings of the host-gut bacteria association analysis mentioned above.

### 3.5. Composition and Functional Potential of Gut Microbes

**Figure 5:**
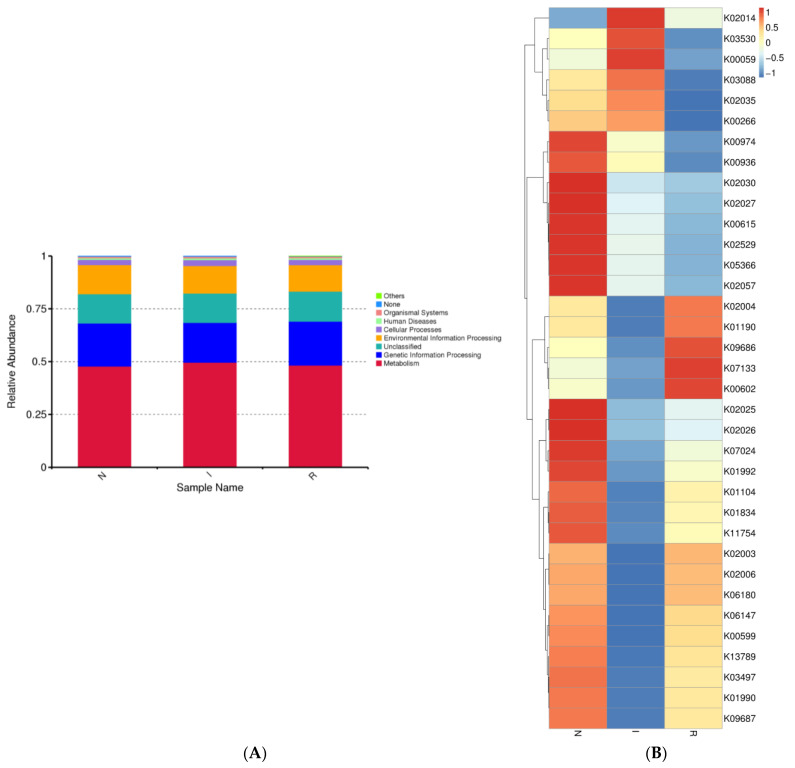
Microbial function prediction of three groups of pig’s gut bacteria. The picture indicated the KEGG functional category. (**A**) shows level 2 of KEGG functional category. (**B**) shows level 3 of KEGG functional category. Abbreviations: N, samples from normal control group; I, samples from the infected group; R, samples from recovery group.

The gut microbes within each group exhibited distinct distributions across various biological pathways. We observed the relative abundance of gut microbes associated with different pathways, noting that the majority of bacterial communities were linked to metabolic pathways across all three groups. Additionally, genetic information processing was the second most correlated pathway in each group (Figure 5A). Moreover, bacterial functional predictions were depicted using a heatmap, considering the top 35 pathways. These pathways encompassed genetic processing as well as the metabolism of lipids, amino acids, and carbohydrates. Group N exhibited notably elevated expression of pathways associated with carbon metabolism and the biosynthesis of amino acids. Conversely, within group I, the most highly expressed pathway pertained to fatty acid metabolism, whereas purine metabolism was the most highly expressed pathway in group R (Figure 5B).

## 4. Discussion

*Escherichia coli* (*E. coli*) is a Gram-negative bacterium commonly found in the intestinal microbiome of different hosts. While typically a commensal inhabitant of the gastrointestinal tract, *E. coli* can also pose health risks by causing intestinal and extraintestinal disorders. However, the majority of commensal strains of *E. coli* rarely cause illness in their hosts [11]. Conversely, strains of *E. coli* expressing specific virulence factors are more likely to induce diarrheal illnesses. Infections in swine are attributed to *Escherichia coli* (*E. coli*), commonly referred to as swine colibacillosis, which can be caused by a wide range of complications [7]. Enterotoxigenic *E. coli* (ETEC) can provoke gastrointestinal ailments, notably contributing to conditions such as PWD, ED, and neonatal diarrhea, usually after weaning [10].

The pig gut hosts a highly intricate and different microbial population that varies among the intestines and is influenced by factors such as age and diet [25]. Microbial colonization of the pig intestine begins at birth and continues to evolve during the weaning phase [26]. Consequently, the microbial composition of newly weaned pigs is particularly sensitive to disruption, potentially exacerbated by the stress of weaning and changes in diet. This increased vulnerability may render pigs more susceptible to colonization by pathogens [27].

The histopathological findings revealed multiple changes in the lamina propria and submucosa of the duodenum and jejunum, as well as in the lamina propria of the ileum, in the infected group compared to the control group. Villous atrophy accompanied by crypt hyperplasia was pervasive across all intestinal segments, with pronounced severity noted in the ileum. Additionally, neutrophil infiltration, mild epithelial lesions, and crypt damage were recorded in our study. These histopathological observations are consistent with previously reported findings of comparable morphological alterations in the small intestine of animals due to enteropathogenic bacterial invasion. These findings collectively underscore the extensive pathological alterations elicited by *E. coli* infection in the small intestine of piglets [28,29].

The Venn diagram depicting operational taxonomic units (OTUs) illustrates the presence of distinct microbial richness within each group, with the *E. coli* diarrheal group exhibiting the highest richness, indicative of substantial biodiversity among groups in this study. This observation is consistent with the results obtained from various alpha diversity parameters, including observed species, Chao1, Shannon diversity, ACE, and PD-whole tree indices [28]. The variability in microbial diversity is influenced by multiple factors, including the dosage load and method of infection [30]. Additionally, age, environmental conditions, and dietary composition also contributed greatly to the variability and microbial diversity among the experimental groups. These findings imply that *E. coli* infection plays a role in shaping and expanding the gut microbiota by promoting growth conditions favorable to certain pathogenic bacteria while suppressing the growth of commensal microbes [18,31].

Principal coordinate analysis (PCoA) confirmed the presence of evident dysbiosis in the gut microbiota following *E. coli*-induced diarrhea. While the normal control and infected groups displayed slight clustering together, the recovery group formed a distinct cluster that was distinctly separate from both groups. Each group exhibited separate clusters in the PCoA plot, confirming the presence of unique microbial compositions within each group. Although there was some resemblance between the normal and infected groups, the recovery group exhibited distinct flora. This observation suggested that the strength of the *E. coli* infection may not have been as pronounced as that reported in chronic infection studies with *E. coli* (Figure 3). This discovery suggested that the pathogenic properties of *E. coli* could alter the microbial composition of the intestines of pigs [28,30].

In this study, we conducted an assessment and comparison of the gut microbiome in healthy piglets, those naturally infected with *E. coli* and those that had recovered from the infection. Our investigation systematically examined the microbial profiles of piglets associated with *E. coli* infection and assessed the bacterial alterations relative to those of their healthy counterparts, as well as those of piglets experiencing diarrheal symptoms and subsequent recovery. A previous study revealed that *Firmicutes* and Bacteroidetes are the dominant phyla in the mammalian gut despite the presence of various influencing factors such as disease, age, breed, diet, and sex [32]. The obtained findings corroborated those of previous studies, indicating that *Bacteroidetes* and *Firmicutes* are the dominant phyla in both healthy and recovered piglets. However, we observed that *Fusobacterota* was the dominant phylum in piglets with *E. coli*-induced diarrhea. This pattern was also observed in other diarrheal diseases [33,34]. The *Bacteroidetes:Firmicutes* ratio observed in the current study diverged from previous findings, potentially attributed to the natural mode of *E. coli* infection or other factors such as age, breed, feeding practices, and environmental conditions [34,35]. Diarrheal piglets exhibited a lower abundance of *Firmicutes*, consistent with prior research [18]. The elevated abundance of *Fusobacterota* in the *E. coli*-infected group confirmed its association with pathogenic conditions and inflammation in the intestines. In contrast, recovered pigs once again demonstrated the dominance of *Firmicutes* and *Bacteroidetes* at the phylum level, indicating their active involvement in carbohydrate metabolism [36,37].

A detailed investigation of the gut microflora at the class level in comparison to previous studies was conducted in this study. Consistent with prior research, we identified the class *Bacteroidia* as dominant in both group N (healthy) and group R (recovered). Conversely, group I (infected) exhibited an abundance of the class *Fusobacteria*, aligning with findings from other diarrheal conditions [38,39]. Additionally, a slight increase in the abundance of the class *Bacilli* in group R was observed, which correlated with improved gut health in this cohort. The increase in *Bacteroidetes* was primarily driven by a greater abundance of the *Muribaculacae* genus, while the greater abundance of *Fusobacterota* was attributed to the relative abundance of the *Fusobacteria* class. In our study, the heat map analysis revealed distinct bacterial composition patterns at the phylum level, with group R displaying significantly greater abundances of *Bacteroidota* and *Fibrobacterota* than the infected group. *Fibrobacteria* are known for cellulose degradation, and their presence in the gut of the recovery group can be attributed to their beneficial effects on gut health [24]. The highly expressed phyla in group I were associated with inflammation and pathogenic conditions in the intestines, whereas group R and group N exhibited highly expressed phyla that are beneficial for gut health.

To thoroughly delineate the differences between the recovery group and the *E. coli*-infected group, a LEfSe analysis of the gut microbial composition of the pigs was conducted. In this study, the taxonomic cladogram and the associated LDA scores facilitated visualization and confirmation of the effects, aligning with other analyses conducted. Notably, group R exhibited dominance of the phyla *Euryarchaeota* and *Bacteroidota*, while group N and group I showed dominance of the phyla *Firmicutes* and *Fusobacteriota*, respectively. Moreover, the LDA scores highlighted the significant expression of the *Muribaculacea* family in group R, which is known for its anti-inflammatory functions [24,40]. Consistent with prior research, our data revealed that the *Lactobacillus* genus is a signature bacterium that differentiates healthy and diarrheal piglets [41,42]. These LEfSe analyses revealed clear differences in the predominant residents of the intestinal microbiota among the three groups of pigs, consistent with the findings of the host-gut bacteria association analysis conducted earlier.

The gut bacteria of all three groups exhibited stronger correlations with the optimization of metabolic functions; carbon metabolism, biosynthesis of amino acids, and fatty acid metabolism were enhanced, which could strengthen the gut’s homeostasis. In addition, the genetic information processing pathway was also enhanced.

## 5. Conclusions

This study highlighted the significant variations in the gut microbiota composition among healthy, *E. coli*-infected, and recovered piglets. Enterotoxigenic *E. coli* (ETEC) strains cause considerable morphological and inflammatory changes in the small intestine, leading to edema, crypt hyperplasia, and villous atrophy. A noticeable shift in microbial diversity was observed, with the *E. coli*-infected group exhibiting a dominance of pathogen-associated phyla such as *Fusobacteriota*, while the recovered group showed a rebound in beneficial bacteria such as Bacteroidota and *Fibrobacterota*. LEfSe analysis confirmed these results, indicating a correlation between microbial composition and intestinal health. These findings underscore the complex correlation between pathogenic bacteria and the gut microbiota, suggesting that restoring beneficial microbes could be key to recovery from *E. coli*-related intestinal diseases in pigs.

## Figures and Tables

**Table 1:** List of primers used in the present study.

Primer Name	Primer Sequence	Annealing Temp	Size	Reference
Forward primer 338F	ACTCCTACGGGGAGGCAGCAG	50 °C	469 bp	[24]
Reverse primer 806R	GGACTACNNGGGGTATCTAAT			

## Data Availability

The raw data supporting the conclusions of this article will be made available by the authors on request.
